# Natural Resource Monitoring of *Rheum tanguticum* by Multilevel Remote Sensing

**DOI:** 10.1155/2014/618902

**Published:** 2014-07-02

**Authors:** Caixiang Xie, Jingyuan Song, Fengmei Suo, Xiwen Li, Ying Li, Hua Yu, Xiaolan Xu, Kun Luo, Qiushi Li, Tianyi Xin, Meng Guan, Xiuhai Xu, Eiji Miki, Osami Takeda, Shilin Chen

**Affiliations:** ^1^Institute of Medicinal Plant Development, Chinese Academy of Medical Sciences, Beijing 100193, China; ^2^Shandong Academy of Agriculture Sciences, Jinan 250100, China; ^3^China Meheco Co., Ltd., Beijing 100061, China; ^4^Botanical Raw Material Research Department, Tsumura & Co., Ibaraki 300-1192, Japan; ^5^Institute of Chinese Materia Medica, China Academy of Chinese Medical Sciences, Beijing 100700, China

## Abstract

Remote sensing has been extensively applied in agriculture for its objectiveness and promptness. However, few applications are available for monitoring natural medicinal plants. In the paper, a multilevel monitoring system, which includes satellite and aerial remote sensing, as well as ground investigation, was initially proposed to monitor natural *Rheum tanguticum* resource in Baihe Pasture, Zoige County, Sichuan Province. The amount of *R. tanguticum* from images is *M* = S*ρ and *S* is vegetation coverage obtained by satellite imaging, whereas *ρ* is *R. tanguticum* density obtained by low-altitude imaging. Only the *R. tanguticum* which coverages exceeded 1 m^2^ could be recognized from the remote sensing image because of the 0.1 m resolution of the remote sensing image (called effective resource at that moment), and the results of ground investigation represented the amounts of *R. tanguticum* resource in all sizes (called the future resource). The data in paper showed that the present available amount of *R. tanguticum* accounted for 4% to 5% of the total quantity. The quantity information and the population structure of *R. tanguticum* in the Baihe Pasture were initially confirmed by this system. It is feasible to monitor the quantitative distribution for natural medicinal plants with scattered distribution.

## 1. Introduction

Remote sensing has been extensively applied in agriculture in recent years. This approach can provide objective, accurate, and timely information on the ecological environment of crops. Many remote sensing applications for medicinal plants are focused on cultivated medicinal plants. By contrast, natural medicinal plants are largely ignored because of their scattering distribution characteristic and small coverage area. A multilevel monitoring system, which includes satellites, aerial remote sensing, and ground investigation was proposed in this paper to monitor natural* Rheum tanguticum* in the Baihe Pasture, Zoige County, Sichuan Province, to determine the quantitative distribution and population structure of* R. tanguticum* in this region.

A number of studies indicate that domestic* R. tanguticum* are mainly distributed in temperate Asian zones, including the Greater Khingan Mountains, Taihang Mountains, Qinling Mountain, Daba Mountains, and Yunnan-Guizhou Plateau [1, 2].* R. tanguticum* is a peculiar Chinese perennial herb that belongs to the Polygonaceae family. This herb mainly thrives in Southern Gansu Province, Northwest Sichuan Province, and Northeast Tibet. These areas are characterized by high altitudes and short frost-free periods.* R. tanguticum* grows slowly in these regions and has rich substances with good qualities. The popular “Xining Rheum” and “Quanshui Rheum” originated from these areas. Recent studies on* R. tanguticum* are mainly focused on ecological suitability, chemical analysis, and pharmacology [3–7]. The demand for* R. tanguticum* as an ingredient for Tibetan medicine, health products, and specialty food has gradually increased [8].* R. tanguticum* in the Qinghai-Tibet Plateau proliferates slowly because of the cold climate and fragile ecological environment. Moreover, the unreasonable excavation of* R. tanguticum* leads to a significant reduction in the quantity of this resource and may even lead to desertification. Determining the updated status of the quantitative distribution and population structure of* R. tanguticum* is necessary for ecological protection and resource development [9]. Thus, the multilevel monitoring system was established to monitor the updated status of* R. tanguticum* [10–12]. Satellite remote sensing and low-altitude remote sensing were initially applied to monitor the quantitative distribution and population structure of natural* R. tanguticum* in the Baihe Pasture. The viability and effectiveness of the multilevel remote system were evaluated by ground-sampled surveys. This paper presented a new technical method and platform for monitoring wild and scattered medicinal plant resources.

## 2. Materials and Methods

### 2.1. Study Area

**Figure 1:**
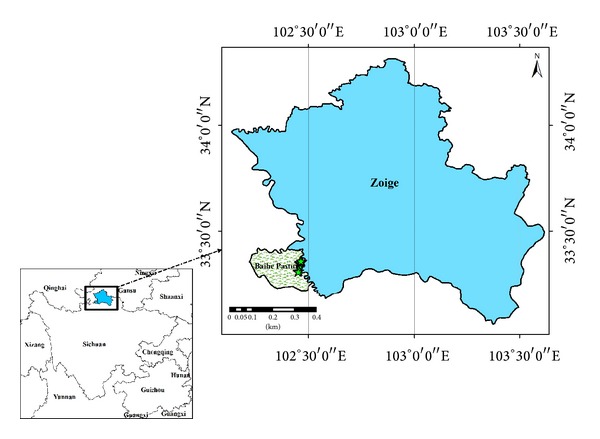
Baihe pasture location (means the fenced field).

Zoige County (102°8′ to 103°39′ E, 33°56′ to 34°19′ N), which is located in the northeastern margin of the Tibetan Plateau, belongs to the northernmost area of Aba, Sichuan Province. This area is bordered by 4 counties of Gansu Province (i.e., Maqu, Luqu, Zhuoni, and Diebu Counties) and Sichuan Province (i.e., Aba, Hongyuan, Songpan, and Jiuzhaigou Counties). Zoige County is an important region of the Northwest Sichuan Pasture and covers an area of 10,620 km^2^ with an altitude of 3,400 m to 3,900 m. Zoige County is located on a plateau with a cold, temperate, and humid monsoon climate with a relative humidity of 68%. The average temperatures in January and July are −9.4 and 11.5°C, respectively. The average annual temperature is 1.7°C, and the highest and lowest temperatures are 25.4 and −29.5°C, respectively. The annual accumulated temperature higher than 10 is 718.4°C. Annual sunshine duration, rainfall, evaporation, gale day, and dust day are 2,506.7 h, 543.2 mm to 761.6 mm, 1,188.24 mm, 11.2 d, and 0.6 d, respectively. Zoige County has abundant water resources and contains several main branches of the Yellow River upstream, including the Heihe, Baihe, and Jiasong Rivers. Zoige County receives significant sunshine and heat energy, thus contributing to its rich 8,084 km^2^ natural grasslands that contain various medicinal plants, including* Fritillaria*,* Cordyceps*,* Gentiana*, Rhubarb,* and Saussurea*. These plants amount to 121 families and 1,094 species.* Rheum palmatum*,* R. tangulicum* and* Rheum officinale* all have distribution in Sichuan Province, but only wild* R. tangulicum* scatters in the studied region, and the other two species are mainly cultivated in farmlands or around farmhouses. The studied region is shown in Figure 1. The studied region is located in Tangke Town, Zoige County, Aba Prefecture, and Sichuan Province with an altitude of 3,437 m and total area of 433 km^2^. Most areas of the Baihe Pasture are covered with natural grasslands, except for the 1,000 ha of crops.

### 2.2. Data Collection

*Satellite Image*. Landsat Thematic Mapper (TM) 5 image with a 0.45 *μ*m to 2.35 *μ*m spectrum range was used to determine the vegetation area in the Baihe Pasture. The image parameters were as follows: N33°25′ E102°14′ image center, blue B1 band, 0.45 *μ*m to 0.52 *μ*m spectrum range, and 30 m resolution; green B2 band, 0.52 *μ*m to 0.60 *μ*m spectrum range, and 30 m resolution; red B3 band, 0.63 *μ*m to 0.69 *μ*m spectrum range, and 30 m resolution; near-infrared B4 band, 0.76 *μ*m to 0.90 *μ*m spectrum range, and 30 m resolution; short-wave infrared B5 band, 1.55 *μ*m to 1.75 *μ*m spectrum range, and 30 m resolution; thermal infrared B6 band, 10.4 *μ*m to 12.5 *μ*m spectrum range, and 120 m resolution; middle-infrared B7 band, 2.08 *μ*m to 3.35 *μ*m spectrum range, and 30 m resolution. TM imaging is suitable for large-scale object monitoring because of its moderate resolution and low cost. The TM5 image shot on 18 July 2008 was chosen in the monitoring of the Baihe Pasture vegetation based on the biological features of natural* R. tanguticum*.

**Figure 2:**
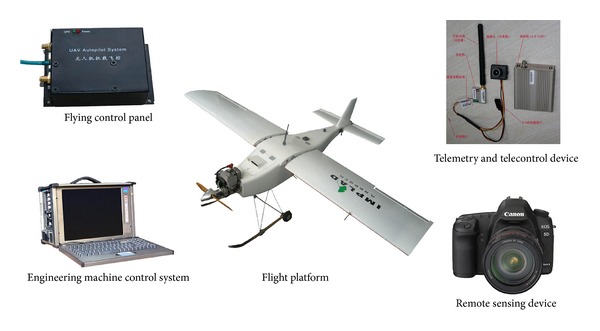
Low-altitude remote sensing system.

The low-altitude remote sensing system consisted of a flight platform, digital camera, single-axis stabilized platform, and equipment control system (Figure 2). The flight platform is an unmanned aerial vehicle from the Institute of Medicinal Plant Development. The Canon 5D digital camera with a charge-coupled device array was adopted for data acquisition. The camera also has array pixels and a lens focal length of 4368 pixels × 2912 pixels and 35 mm, respectively. The 0.1 m resolution of the camera for aerial images was appropriate for the requirements of the survey goal based on the ground survey. The high-resolution aerial photography for wild* R. tanguticum* was initially conducted in the Baihe Pasture of Zoige County by the low-altitude remote sensing system.

*Ground Survey*. A total of 10 fenced zones with an area of 200 m × 200 m were selected for the* R. tanguticum* field survey. Four 10 m × 10 m plots were established in the 10 fenced zones based on the plot-setting principle. The ground survey for* R. tanguticum* was conducted separately in the fenced zones in July 2008, 2010, and 2011.

### 2.3. Study Method

**Figure 3:**
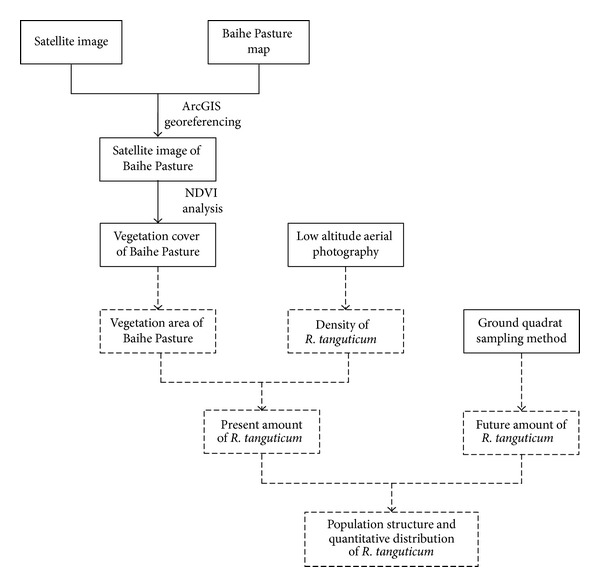
Overall technical route.

The complete technical road map is summarized in Figure 3. First, the location and boundary of the Baihe Pasture in the satellite image were determined by using ArcGIS software based on the Landsat TM and Baihe Pasture map. Second, the interpretation keys of the vegetation zone were established by normalized difference vegetation index (NDVI) analysis based on the Landsat TM image. Third, ground samples were designed based on the topography and geomorphology of the Baihe Pasture. The distribution density of* R. tanguticum* in vegetation was determined by using low-altitude images with 0.1 m resolutions. Finally, the amount of natural* R. tanguticum* resource was calculated based on the vegetation area from the satellite image and the* R. tanguticum* distribution density from low-altitude remote sensing. The results were compared and assessed with those of the ground survey.

## 3. Data Analysis

### 3.1. Image Processing

**Figure 4:**
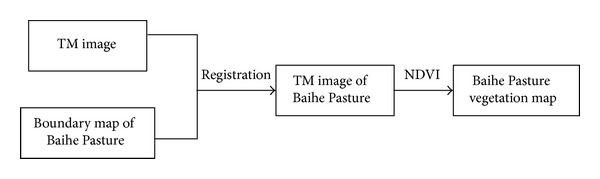
Image processing.

Bands 4, 3, and 2 in the TM image were fused to distinguish the vegetation and water. The Baihe Pasture boundary in the TM image was determined via the registration function by the feature points. Thus, the Baihe Pasture satellite image was detached from the original TM image. NDVI is an internationally accepted criterion for vegetation coverage level. Consider that NDVI = (NIR −* R*)/(NIR +* R*), where NIR denotes the near-infrared band reflectivity and* R* stands for red band reflectivity. An NDVI value in the range of zero to one generally refers to vegetation regions. The vegetation region of the Baihe Pasture was determined based on the NDVI analysis in Figure 4.

### 3.2. Object-Based Image Classification

High-spatial resolution images have limited spectral information but contain many spatial information of objects, such as size, shape, and topological information. The object-oriented approach classifies different objects based on spectral signature, shape, and contextual relationship. Homogeneous objects were initially formed by multiscale image segmentation. Thereafter, image classification was used to endow the objects with different semantic information based on size, spectrum, and shape parameters. These objects were then used as a basis for the fuzzy classification of the image. The use of spectral, textural, and shape properties, as well as fuzzy thinking, might reduce uncertainty in the classification process. The results of the object-based image interpretation showed better integration levels than the pixel-based interpretation method [9, 13, 14], which is suitable for high-spatial resolution images.

## 4. Results

### 4.1. Biological and Community Characteristics of* R. tanguticum*

**Figure 5:**
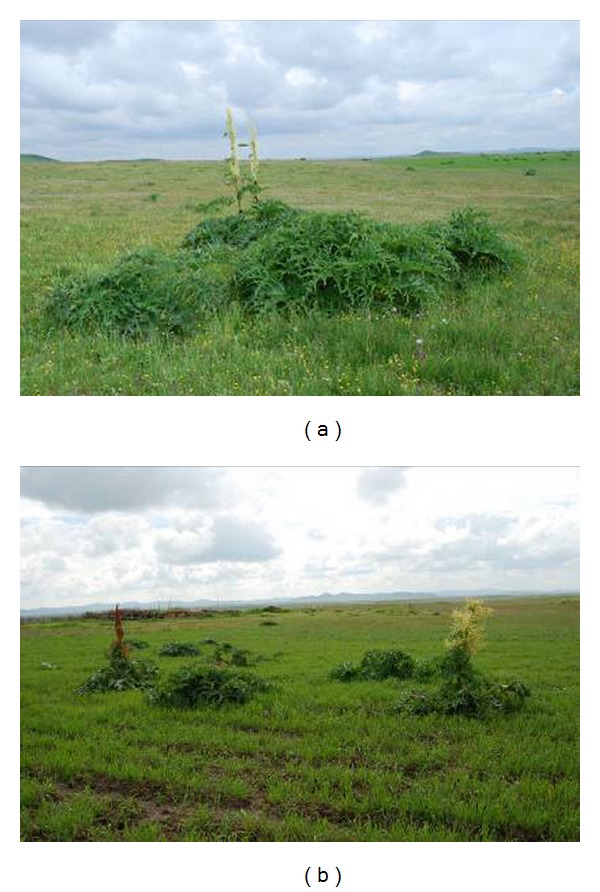
Community characteristics of wild* R. tanguticum*.

*R. tanguticum* is a perennial and tall herb that is resistant to cold but has zero endurance to high temperatures. This herb thrives in a cool and moist climate and is mainly scattered in forests, scrubs, and meadows. Natural* R. tanguticum* plants in the Baihe Pasture can grow for approximately seven or eight years with a height of 1.5 m to 2.0 m and a corolla diameter of 2.4 mm to 3.7 mm. The blooming stage of* R. tanguticum* is from May to July. The ecology community type of* R. tanguticum* is alpine meadows with only an herb layer. The vegetation coverage of such alpine meadows is more than 95% and includes a small amount of cultivated barley.  Figure 5 presents the community characteristics of* R. tanguticum*.

**Figure 6:**
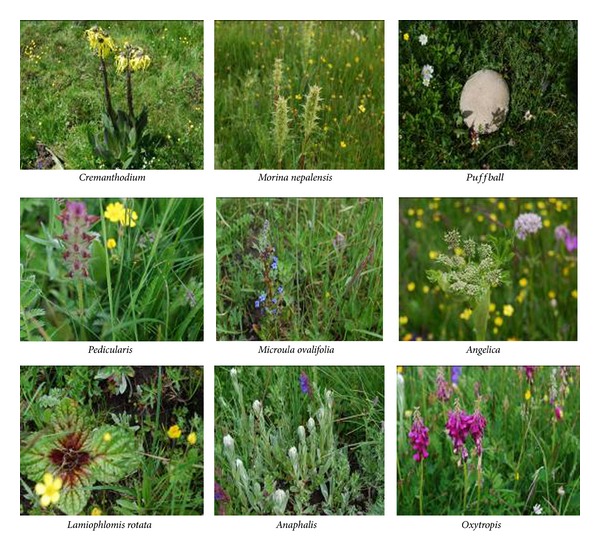
Accompanying plant picture of wild* R. tanguticum*.

The accompanying plants of Rhubarb are mainly Ranunculaceae, Cyperaceae, Gramineae, Rosaceae, Compositae, Polygonaceae, Dipsacaceae, Leguminosae, Umbelliferae, Gentianaceae, Labiatae, Boraginaceae, Basidiomycetes, and others. Cyperaceae and Gramineae assume absolute superiority among these plants. Ranunculaceae, Compositae, and a few fungi compose the weed layer.  Figure 6 and Table 1 show the vegetation type and accompanying plants of natural* R. tanguticum* in the Baihe Pasture, respectively.

### 4.2. Multilevel Remote Sensing Resource Monitoring for* R. tanguticum*

Based on NDVI anlysis, the pixel numbers of 0.1 < NDVI < 1 and NDVI < 0.1 were 305,676 and 189,658, with each representing the vegetation and nonvegetation regions, respectively. Therefore, the vegetation area ratio (*P*), area (*M*), and vegetation area (*M*_1_) of the Baihe Pasture were *P* = 305676/(305676 + 189658) = 62%, *M* = (305676 + 189658) × 30 m × 30 m = 445 km^2^ (44,500 ha), and *M*_1_ = 445 × 62% = 276 km^2^ (27,600 ha), respectively. The results were consistent with the 43,333 ha provided by the Baihe Pasture. Therefore, remote sensing technology was feasible in monitoring the fields.

As a matter of experience, only* R. tanguticum* with more than 1 m^2^ of coverage (called effective resource) could be interpreted from the image. Thus, the average density of available* R. tanguticum* could be calculated by the interpretation results of many low-altitude images. The effective resources of* R. tanguticum* in the entire Baihe Pasture were that the Baihe Pasture vegetation area multiplied by the average* R. tanguticum* density, which was equal to 350,000. Therefore, the amount of available natural* R. tanguticum* resources in the Baihe Pasture in 2008 was 350,000.

### 4.3. *R. tanguticum *Sample Investigation

Ten vegetation fences, each with an area of 200 m × 200 m, except for the third fence with 150 m × 150 m, were established in the Baihe Pasture, and there were four 10 m × 10 m quadrats in every fence. Ground survey work was conducted in July 2008, 2010, and 2011 in the above fences and quadrats (Table 2).

The ground survey results in 2008 indicated the existence of 2.6* R. tanguticum* per 100 m²; thus, the total amount of* R. tanguticum* in the Baihe Pasture was *M*_1_(276 km^2^) × 2.6/100 m² = 718 × 10^4^.

The ground survey results in 2010 indicated that the number of* R. tanguticum* in each fence was approximately 1,520, except for approximately 855 in the third fence. Thus, approximately 3.8* R. tanguticum* per 100 m² existed, and the total amount of* R. tanguticum* was 1,049 × 10^4^.

The results in 2011 indicated that the average number of* R. tanguticum* in each fence was approximately 1,220; the minimum amount was approximately 300 in the second fence, and the amount was approximately 1600 in the first, fourth, and sixth fences. The calculation result was 3.1* R. tanguticum* per 100 m². Thus, the total amount of* R. tanguticum* in 2011 was 856 × 10^4^.

Quantity variance existed in the total amount of* R. tanguticum* based on the results of different years because of operation errors and other factors. However, the total quantity of* R. tanguticum* was approximately 750 × 10^4^ to 1,000 × 10^4^. The current available amount and the future total amount of natural* R. tanguticum* resources in 2008 were, respectively, 35 × 10^4^ and 718 × 10^4^ based on the results of the multilevel remote sensing and ground survey. The population structure of natural* R. tanguticum*, that is, the available amount of* R. tanguticum*, comprised 4% to 5% of the total amount. Therefore, the safe digging quantity of* R. tanguticum* in the Baihe Pasture was 4% to 5% for the protection of natural resources.

## 5. Discussion

Remote sensing technology has become an important means of obtaining geographic environment information. This technology has remarkably extended and improved the ability of humans to recognize their surrounding environment. Technology innovation in remote sensing provides a new method and platform for surveying natural Chinese medicine resources

(1) Remote sensing has been extensively applied in agriculture in recent years [15–25]. Remote sensing technology can provide objective, accurate, and timely information on the ecological environment of crops and is an important data source for precision agriculture [26]. However, a significant difference exists in the resource distribution between agricultural and natural Chinese medicinal herbs. Artificially cultivated crops are characterized by large areas, centralized distributions, and regular shapes. Thus, these crops have significant image features that are easy to interpret. By contrast, natural Chinese medicinal herbs often exist in wide, inaccessible regions and are scattered in different vegetation communities. Thus, interpreting natural Chinese medicinal herbs from satellite images was difficult because of the lack of distinct image features. Multilevel remote sensing was initially proposed to monitor the natural* R. tanguticum* in the Baihe Pasture. Based on the satellite image results, the Baihe Pasture area was 445 km^2^ (44,500 ha), which was consistent with the 43,333 ha provided by the administrative department of the Baihe Pasture. The vegetation coverage in the Baihe Pasture was 276 km^2^.* R. tanguticum* density was 2.6 to 3.8 per 100 m² based on the result of the multilevel remote sensing. Compared with the ground investigation, the multilevel remote sensing technology system was fast and convenient. Results indicated that the available amount of* R. tanguticum* comprised 4% to 5% of the total amount. The quantity information and population structure of* R. tanguticum* in the Baihe Pasture of Zoige County were initially confirmed by this multilevel monitoring system. This system was also successfully applied in monitoring* Ferula sinkiangensis* in the fourth survey of the Chinese medicine resources of Xinjiang.

(2) An object-oriented image-recognition method was initially adopted to extract* R. tanguticum* in low-altitude aerial images. Only* R. tanguticum* with coverage greater than 1 m^2^ could be extracted from the low-altitude aerial image because of the spatial resolution limitations. Therefore, the amount of* R. tanguticum* obtained from multilevel remote sensing was the current available resources, whereas the results from the ground investigation were the total resources because all* R. tanguticum* sizes could be observed through ground surveys. The amount of* R. tanguticum* obtained from the ground survey represented the total available resource in three years to five years. The results from the two methods (remote sensing, ground survey) represented the present and the future amounts of* R. tanguticum*. Thus, the corresponding method should be selected to satisfy different demands and purposes.

(3) The optimum time for monitoring* R. tanguticum* through remote sensing was early July because* R. tanguticum* was in bloom and taller than the surrounding vegetation during this period. The ground survey results in three years indicated that the total quantity of* R. tanguticum* was approximately 750 × 10^4^ to 1,000 × 10^4^. The available amount of natural* R. tanguticum* resources was 35 × 10^4^ in 2008 based on the results of the multilevel remote sensing. However, the total amount in 2008 was 718 × 10^4^ based on the ground survey results. So, the safety digging quantity of* R. tanguticum* in the Baihe Pasture was 4% to 5% for the protection of natural resources.

We conclude that the use of low-altitude remote sensing technology to monitor the scattered distribution of natural medicinal plants was feasible. This study provided a new technical system for monitoring objects with scattered distributions.

## Figures and Tables

**Table 1:** Accompanying plants of wild *R. tanguticum*.

Species name	Family	Genus	Remarks
*Anaphalis flavescens. *	Asteraceae	*Anaphalis *	Weed layer
*Aster tongolensis *		*Aster *	Weed layer
*Carpesium lipskyi *		*Carpesium *	Weed layer
*Ligularia virgaurea *		*Saussurea *	Weed layer
*Leontopodium longifolium *			Weed layer
*Saussurea graminea *		*Ligularia *	Weed layer
*Saussurea superba *		*Trollius *	Weed layer
*Trollius ranunculoides *		*Leontopodium *	Weed layer

*Thlaspi arvense *	Brassicaceae	*Thlaspi *	Weed layer

*Blysmus sinocompressus *	Cyperaceae	*Kobresia *	Dominant population
*Kobresia pygmaea *			Dominant population
*Kobresia setchwanensis*			Dominant population
*Kobresia humilis *			Dominant population
*Kobresia tibetica *			Dominant population
*Kobresia kansuensis *		*Blysmus *	Dominant population

*Astragalus polycladus *	Fabaceae	*Gueldenstaedtia *	Weed layer
*Gueldenstaedtia diversifolia *		*Oxytropis *	Weed layer
*Oxytropis kansuensis*			Weed layer
*Oxytropis ochrocephala *		*Astragalus *	Weed layer

*Agrostis schneideri *	Gramineae	*Elymus *	Dominant population
*Cymbopogon distans *		*Roegneria *	Dominant population
*Deyeuxia scabrescens *		*Festuca *	Dominant population
*Deschampsia caespitosa *		*Poa *	Dominant population
*Elymus nutans *			Dominant population
*Festuca ovina *		*Deyeuxia *	Dominant population
*Heteropogon contortus *		*Deschampsia *	Dominant population
*Koeleria cristata *		*Agrostis *	Dominant population
*Miscanthus sinensis *		*Koeleria *	Dominant population
*Poa pachyantha *		*Heteropogon *	Dominant population
*Poa pratensis *		*Cymbopogon *	Dominant population
*Roegneria nutans *		*Miscanthus *	Dominant population
*Stipa capillacea *		*Stipa *	Weed layer
*Stipa purpurea *			Weed layer
*Stipa przewalskii *			Weed layer

*Geranium pylzowianum *	Geraniaceae	*Geranium *	Weed layer

*Polygonum taquetii *	Polygonaceae	*Polygonum *	Weed layer
*Polygonum viviparum *			Weed layer

*Fragaria orientalis *	Rosaceae	*Potentilla *	Weed layer
*Potentilla anserina *			Weed layer
*Potentilla griffithii *		*Fragaria *	Weed layer

*Anemone rivularis *	Ranunculaceae	*Ranunculus *	Weed layer
*Ranunculus tanguticus *		Anemone	Weed layer
*Trollius lilacinus *		*Trollius *	Weed layer
*Trollius ranunculoides *			Weed layer

*Galium verum *	Rubiaceae	*Galium *	Weed layer

*Stellera chamaejasme *	Thymelaeaceae	*Stellera *	Weed layer

*Viola biflora *	Violaceae	*Viola *	Weed layer

**Table 2:** *R. tanguticum* number of ground sample investigation.

Fences	Quadrats Year											
	1 (10 × 10 m²)			2 (10 × 10 m²)			3 (10 × 10 m²)			4 (10 × 10 m²)		
	2008	2010	2011	2008	2010	2011	2008	2010	2011	2008	2010	2011
I (200 × 200 m²)	0	0	2	4	6	7	6	10	5	1	2	1
II (200 × 200 m²)	2	6	1	1	2	0	3	5	0	4	8	2
III (150 × 150 m²)	1	4	0	1	3	4	2	1	4	0	3	1
IV (200 × 200 m²)	2	2	0	2	0	12	0	0	4	1	1	1
V (200 × 200 m²)	3	5	1	3	6	1	3	3	6	5	8	6
VI (200 × 200 m²)	10	18	0	3	4	5	1	2	5	1	0	4
VII (200 × 200 m²)	4	7	1	4	5	5	6	10	3	3	1	4
VIII (200 × 200 m²)	0	0	4	0	0	0	0	0	1	0	0	3
IX (200 × 200 m²)	3	3	4	5	4	2	8	13	4	3	3	3
X (200 × 200 m²)	2	3	3	2	1	4	2	1	4	3	2	5

## References

[1] Xue GJ, He L, Pan X (1995). Development and utilization of research on Chinese Rhubarb. *China Natural Plant Resource*.

[2] Xie ZQ Ecogeographical distribution of the species from *Rheum* L., (Polygonaceae) in China.

[3] Yu H, Xie C, Song J, Zhou Y, Chen S (2010). TCMGIS-II based prediction of medicinal plant distribution for conservation planning: a case study of *Rheum tanguticum*. *Chinese Medicine*.

[4] Suo FM, Song JY, Chen SL (2010). AFLP analysis on genetic relationship among *Rheum tanguticum*, *Rheum palmatum*, and *Rheum officinale*. *Chinese Traditional and Herbal Drugs*.

[5] Liu Y, Wang Q, Jiang M, Li H, Zou M, Bai G (2012). Screening of effective components for inhibition of tyrosinase activity in rhubarb based on spectrum-efficiency-structure-activity relationship. *Chinese Traditional and Herbal Drugs*.

[6] Xie CX, Suo FM, Zhou YQ (2011). Quantitative study on ecological suitability of Chinese herbalmedicine based on GIS. *China Journal of Chinese Materia Medica*.

[7] Chen SL (2011). *Ecology Suitability Regionalization of Chinese Medical Plants*.

[8] Li JP, Chen GS, Lu XF (2007). Status of *Rheum tanguticum* maxim. exbalf resources of Qinghai and countermeasures for protection. *Qinghai Prataculture*.

[9] Wang JF, Bao ST (2006). Application of object-oriented interpretation to information classification on remote sensing image. *Tropical Geography*.

[10] Xie CX, Chen SL, Wang Y, Zhou YQ, Li Y (2008). Some key problems of quantitative estimation in multilevel remote sensing monitoring system of plant medicinal materials. *China Journal of Chinese Materia Medica*.

[11] Xie CX, Chen SL, Lin ZJ (2007). Application of Unmanned Aerial Vehicles to medical plants survey. *Modern Chinese Medicine*.

[12] Zhou YQ, Chen SL, Zhao RH, Xie CX, Li Y (2008). Study on application of low altitude remote sensing to Chinese herb medicinal sustainable utilization. *China Journal of Chinese Materia Medica*.

[13] Zeng J, Li XX, Wang T (2011). Study on object-oriented extracting water from high resolution remote sensing image. *Jiangxi Science*.

[14] Wang QL (2008). *Study on Object-Oriented Remote Sensing Image Classification and Its Application*.

[15] Ahamed T, Tian L, Zhang Y, Ting KC (2011). A review of remote sensing methods for biomass feedstock production. *Biomass and Bioenergy*.

[16] Xiang H, Tian L (2011). An automated stand-alone in-field remote sensing system (SIRSS) for in-season crop monitoring. *Computers and Electronics in Agriculture*.

[17] Zhang YS, Feng R, Ji RP, Chen P, Zhang S, Wu J Application of remote sensing technology in crop chilling injury monitoring.

[18] Anbarashan M, Parthasarathy N (2013). Diversity and ecology of lianas in tropical dry evergreen forests on the Coromandel Coast of India under various disturbance regimes. *Flora: Morphology, Distribution, Functional Ecology of Plants*.

[19] Musa HD, Shaib B (2010). Integrated remote sensing approach to desertification monitoring in the crop-rangeland area of Yobe State. *Nigeria. Journal of Sustainable Development in Africa*.

[20] Huang Q, Zhang L, Wu W, Li D MODIS-NDVI-based crop growth monitoring in China Agriculture Remote Sensing Monitoring System.

[21] Wasige JE, Groen TA, Smaling E, Jetten V (2012). Monitoring basin-scale land cover changes in Kagera Basin of Lake Victoria using: ancillary data and remote sensing. *International Journal of Applied Earth Observation and Geoinformation*.

[22] Yuping M, Shili W, Li Z (2008). Monitoring winter wheat growth in North China by combining a crop model and remote sensing data. *International Journal of Applied Earth Observation and Geoinformation*.

[23] Tao F, Yokozawa M, Zhang Z, Xu Y, Hayashi Y (2005). Remote sensing of crop production in China by production efficiency models: Models comparisons, estimates and uncertainties. *Ecological Modelling*.

[24] Lobell DB, Asner GP, Ortiz-Monasterio JI, Benning TL (2003). Remote sensing of regional crop production in the Yaqui Valley, Mexico: estimates and uncertainties. *Agriculture, Ecosystems and Environment*.

[25] Ahamed T, Tian L, Jiang Y, Zhao B, Liu H, Ting KC (2012). Tower remote-sensing system for monitoring energy crops; image acquisition and geometric corrections. *Biosystems Engineering*.

[26] Lausch A, Pause M, Merbach I (2013). A new multiscale approach for monitoring vegetation using remote sensing-based indicators in laboratory, field, and landscape. *Environmental Monitoring and Assessment*.

